# Phytochemical Analysis and Chymotrypsin Inhibitory Potential of *Galium* sp. and *Solidago* sp. via Effect-Directed HPTLC Bioassay

**DOI:** 10.3390/molecules30132746

**Published:** 2025-06-26

**Authors:** Bartosz Rył, Izabela Jasicka-Misiak

**Affiliations:** Institute of Chemistry, University of Opole, 48 Oleska Str., 45-052 Opole, Poland; izajm@uni.opole.pl

**Keywords:** HPTLC-DPPH^•^, HPTLC-chymotrypsin inhibition, antioxidant activity, *Galium aparine* L., *Galium verum* L., *Solidago virgaurea* L., *Solidago canadensis* L.

## Abstract

Chymotrypsin inhibitors were initially considered mainly as anti-nutritional factors. However, the potential for their use as therapeutics has been recognized, particularly in the control of cancer, neurodegenerative diseases, and inflammatory processes. The search for new, effective, and safe chymotrypsin inhibitors has become important not only for food and feed safety reasons, but also in the search for new compounds with potential for use in the pharmaceutical industry. Oxidative stress is also an integral etiological factor in the development of the aforementioned pathological conditions. Antioxidants supplied with food can have an impact on reducing the probability of developing these diseases. Herbaceous plants are a valuable reservoir of biologically active chemical compounds, which can show both inhibitory effects against a number of enzymatic reactions and have antioxidant activity. The compounds found within them are also often characterized by higher bioavailability and safety than their synthetic analogs. In the present study, phytochemical characterization of plant materials *Galium aparine* L., *Galium verum* L., *Solidago virgaurea* L. and *Solidago canadensis* L. was performed, in order to search for new, potential substances with chymotrypsin inhibitor and antioxidant properties. Antioxidant and inhibitory activities against chymotrypsin were determined using effect-directed HPTLC. The total content of phenolic compounds and flavonoids and antioxidant activity were also determined in UV-Vis spectrophotometric tests. Both plant species showed antioxidant and chymotrypsin inhibitory activity. Among the methanol and methanol:water extracts, the extracts from *Solidago* sp. showed stronger inhibitory and antioxidant activity. However, in the case of dichloromethane extracts, *Galium aparine* inhibited chymotrypsin activity in a stronger manner than *Solidago* sp. The results indicate the application potential of compounds obtained from these plants as chymotrypsin inhibitors and antioxidant agents.

## 1. Introduction

Serine proteases belong to the best-studied group of proteases [1], enzymes of the hydrolase class that play an important role in diverse biological processes, including immune processes, regulation of metabolism and digestion, blood coagulation, and are associated with disease pathogenesis, including the process of cancer formation, the development of rheumatic diseases, and wound healing [2]. For a long time, chymotrypsin inhibitors were referred primarily as anti-nutritional agents, but, over time, the potential for their therapeutic use has been recognized [3]. They may be applied as immunotherapeutic, anticancer, anti-inflammatory, and antiviral agents, and in the treatment or prevention of Alzheimer’s disease [4,5,6]. Therefore, it is important to strictly control the activity of serine proteases and search for safe, bioactive molecules that exhibit inhibitory activity against chymotrypsin.

Oxidative stress also plays an important role in the development of a number of diseases, for example, cardiovascular diseases, cancer, respiratory diseases, autoimmune diseases (rheumatoid arthritis, systemic lupus erythematosus, multiple sclerosis), type 2 diabetes, and neurological and neurodegenerative diseases (including Alzheimer’s, Parkinson’s and Huntington’s diseases) [7,8,9]. Oxidative stress is led by a disruption in the ratio of reactive oxygen species (ROS) and reactive nitrogen species (RNS) produced in the body and the antioxidants available to neutralize them that are produced endogenously and taken in with the diet [10]. Valuable sources of exogenous antioxidants are compounds of plant origin, belonging to the classes of carotenoids, alkaloids, and phenolic compounds (phenolic acids, hydroxybenzoic acids, flavonoids, anthraquinones, coumarins and tannins, among others) [11].

Plants thus represent a valuable reservoir of structurally diverse bioactive compounds [12]. They can exhibit both antioxidant [13] and inhibitory effects against chymotrypsin and other serine proteases [14]. Plant extracts are complex mixtures of compounds that are interesting sources of active substances, combined with a matrix composed of lipids, sugars, proteins and other compounds that can act as an interfering factor in the analysis. Effect-directed (high-performance) thin-layer chromatography (ED-TLC/HPTLC) is a combination of planar chromatography and chemical/biological/biochemical analysis focused on detecting the desired direction of activity of analytes included in complex mixtures [15]. The minimal sample preparation requirements for analysis [16], coupled with relatively small equipment requirements and the ability to analyze more than a dozen samples at the same time, make ED-HPTLC a rapid and relatively inexpensive technique. The ability to simultaneously separate, qualitatively identify, and determine biological activity in in vitro effect-directed assays—including antioxidant, antimicrobial and enzyme inhibition of complex samples in situ [17,18]—in a short period of time has made effect-directed analysis in HPTLC popular in the search for new bioactive compounds. Current analytical techniques also allow further identification of compounds from active zones by hyphenation to MS (mass spectrometry) [19].

The purpose of the present study was to characterize the phytochemicals (total phenolics content, total flavonoids content, antioxidant activity) of extracts from *Galium aparine* L., *Galium verum* L., *Solidago virgaurea* L. and *Solidago canadensis* L. and to determine their inhibitory activity against chymotrypsin and their antioxidant profile using enzyme-inhibition-HPTLC and HPTLC-DPPH^•^ tests. Researchers from around the world who study the biological activity of plants are increasingly turning to extremely exotic plants. In this study, plant species commonly found in Europe and Asia were selected and analyzed using innovative techniques for analyzing biological activity. The activity of the native medicinal plant *Solidago virgaurea* was also compared with that of the invasive *Solidago canadensis*. Numerous compounds belonging to the classes of phenolic acids, flavonoids and iridoids, among others, have been identified in these plant species to date. Despite belonging to different families—*Galium* sp. belongs to the *Rubiaceae* family, while *Solidago* sp. belongs to the *Asteraceae* family—chlorogenic acid, ferulic acid, caffeic acid, catechins, quercetin and rutin have been detected in all analyzed species [20,21,22,23,24,25,26,27,28,29].

## 2. Results

### 2.1. Extracts Yield

The amounts of extracts obtained after the process of extracting 5 g of raw material, evaporation of solvent, and freeze-drying are shown in Table 1.

### 2.2. Total Phenolics Content (TPC), Total Flavonoids Content (TFC), and Antioxidant Activity

The results of the analysis of basic phytochemical parameters—total phenolic content (TPC), total flavonoid content (TFC) and antioxidant activity—in DPPH^•^ radical, ABTS^+•^ cation radical and Ferric Reducing Antioxidant Power (FRAP) assays are presented in Table 2.

The total phenolics content of methanol:water extracts (MeOH:H_2_O, 70:30 *v*/*v*) was higher than methanolic extracts. The highest TFC value was obtained for lyophilized *S. virgaurea* (*Sv* L) MeOH:H_2_O and was 200.53 ± 4.48 mg gallic acid equivalent per gram of crude extract (mg GAE/g). The highest TPC among methanolic extracts was also determined for *Sv* L and was 184.89 ± 6.11 mg GAE/g. Commercial *Galium aparine* plant material (*Ga* C) extracts showed the lowest TPC value, with 34.49 ± 1.12 mg GAE/g for the methanolic extract and 37.18 ± 0.00 mg GAE/g for the MeOH:H_2_O extract. The complete results of TPC determination are shown in Table 2.

As for the determination of total flavonoids, higher values were obtained for methanolic extracts than for methanol:water extracts. The highest TFC value was presented by the lyophilized *G. verum* (*Gv* L) MeOH extract, with 6.32 ± 0.16 mg rutin equivalent per gram of crude extract (mg RE/g). The lowest value among methanolic extracts, however, was obtained for lyophilized *G. aparine* (*Ga* L) and was 0.74 ± 0.13 mg RE/g. Among MeOH:H_2_O extracts, the highest value was achieved for lyophilized *S. canadensis* (*Sc* L) and was 3.93 ± 0.11 mg RE/g, and the lowest for commercial *G. aparine* (*Ga* C), with a result of 0.16 ± 0.04 mg RE/g. The complete results of TFC determination are shown in Table 2.

The results of total antioxidant activity determinations in the DPPH^•^ assay indicate the highest activity of the methanol:water extract from *Sv* L at 285.22 ± 4.22 mg Trolox equivalent per gram of crude extract weight (mg TE/g). The weakest activity among MeOH:H_2_O extracts was shown by *Ga* C extract, with an activity of 50.76 ± 2.96 mg TE/g. Among the methanolic extracts, the strongest and weakest activity was that of extracts from the same plants—a value of 163.47 ± 3.72 mg TE/g was achieved for *Sv* L MeOH, while a value of 33.73 ± 3.57 mg TE/g was achieved for *Ga* C MeOH. Due to the suitability of the DPPH^•^ test for evaluating the antioxidant activity of lipophilic compounds, determinations were also performed for dichloromethane extracts. The lyophilized *G. aparine* (*Ga* L) DCM extract had the strongest activity, at 27.07 ± 0.93 mg TE/g, while the commercial *G. verum* (*Gv* C) DCM extract had the weakest, at 15.33 ± 0.35 mg TE/g. The complete results of antioxidant activity in the DPPH^•^ radical test are shown in Table 2.

The FRAP test also showed the strongest antioxidant activity for extracts of freeze-dried *Solidago virgaurea* plant material. The activity of the MeOH:H_2_O extract was 294.84 ± 6.11 mg Trolox equivalent per gram of crude extract (mg TE/g). Methanolic extract from this material had an activity of 183.13 ± 8.00 mg TE/g. The lowest antioxidant activity result was achieved, as in the DPPH^•^ test, for extracts from the commercial material of *Galium aparine*. The activity of the *Ga* C MeOH:H_2_O extract was 18.13 ± 0.51 mg TE/g, and that of the MeOH extract was 17.48 ± 0.28 mg TE/g. The complete results of antioxidant activity in FRAP test are shown in Table 2.

Similar results were obtained in the ABTS^+•^ cation radical test. The strongest activity was obtained for *Sv* L MeOH:H_2_O extract: a value of 249.39 ± 9.34 mg of Trolox equivalent per gram of crude extract (mg TE/g). Among methanol:water extracts, *Ga* C MeOH:H_2_O extract had the lowest activity. Among methanolic extracts, the highest activity was obtained for *Sv* C: a value of 149.85 ± 4.55 mg TE/g. Comparable activity was shown for freeze-dried *Solidago virgaurea* extract: a value of 142.29 ± 1.98 mg TE/g. However, *Ga* C extract showed the lowest activity among the methanolic extracts, at 32.71 ± 3.43 mg TE/g. The ABTS^+•^ cation radical test also allows the analysis of dichloromethane extracts. The highest value of antioxidant activity among these extracts was obtained for *Ga* L plant material (27.96 ± 0.37 mg TE/g), and the lowest for *Gv* C (11.39 ± 0.93 mg TE/g). The complete results of antioxidant activity in the ABTS^+•^ cation radical test are shown in Table 2.

### 2.3. HPTLC-DPPH^•^

**Figure 1:**
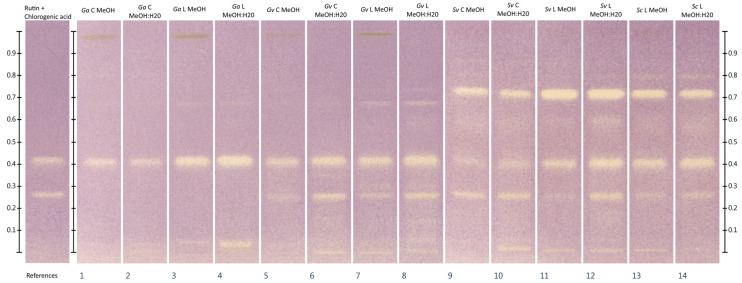
Results of HPTLC-DPPH^•^ analysis of methanolic and methanol:water extracts. Standards were applied at volume of 3 µL, *Galium* sp. extracts 3 µL, *Solidago* sp. 2.5 µL. MeOH—methanolic extracts, MeOH:H_2_O—extracts prepared in methanol:water mixture (70:30 *v*/*v*), *Ga*—*Galium aparine* L., *Gv*—*Galium verum* L., *Sv*—*Solidago virgaurea* L., *Sc*—*Solidago canadensis* L., C—extracts from commercially available plant material, L—extracts from freeze-dried plant material self-collected from wild sites. Stationary phase: HPTLC silica gel 60 plates on aluminum support (20 × 10 cm). Development conditions: first development in MP 2.1: ethyl acetate:methyl ethyl ketone:methanol:diethyl ether:water:formic acid (30:30:10:10:10:10 *v*/*v*/*v*/*v*/*v*/*v*) at distance of 5.5 cm from application zone, second development in MP 2.2: chloroform:ethyl acetate:formic acid (40:10:1 *v*/*v*/*v*) to the height of the target solvent front position (70 mm from the application line). Derivatization agent: DPPH*^•^* methanolic solution (2.5 mM).

**Figure 2:**
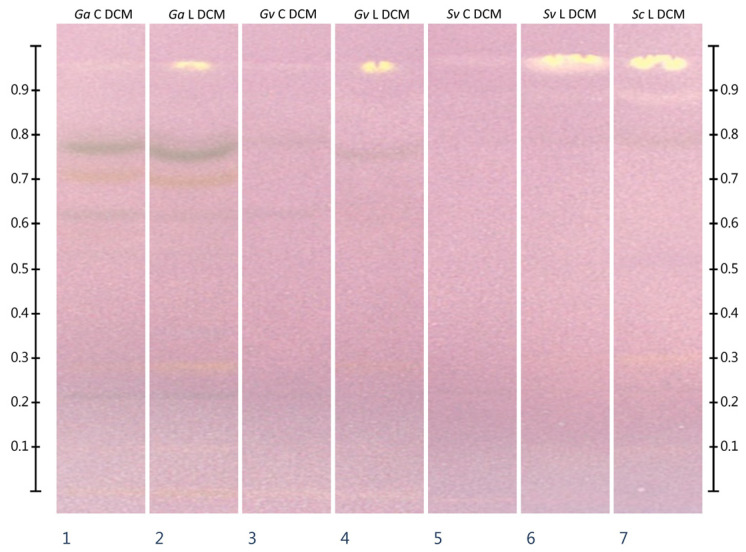
Results of HPTLC-DPPH^•^ analysis of dichloromethane extracts. Samples were applied at volume of 15 µL. DCM—extracts prepared in dichloromethane, *Ga*—*Galium aparine* L., *Gv*—*Galium verum* L., *Sv*—*Solidago virgaurea* L., *Sc*—*Solidago canadensis* L., C—extracts from commercially available plant material, L—extracts from freeze-dried plant material self-collected from wild sites. Stationary phase: HPTLC silica gel 60 plates on aluminum support (20 × 10 cm). Development conditions: MP 1: ethyl acetate:toluene:formic acid (80:20:2 *v*/*v*/*v*) at distance of 7 cm from the application zone. Derivatization agent: DPPH^•^ methanolic solution (2.5 mM).

Dichloromethane extracts showed negligible antioxidant activity in the HPTLC-DPPH^•^ assay. Extracts from freeze-dried plant materials showed one intense zone of antioxidant activity at an Rf of about 0.96, in contrast to commercial extracts, which showed a barely detectable DPPH^•^ color change in this zone. In the case of methanolic and methanol:water extracts, the fewest number of inhibition zones was observed in *Galium aparine* extracts. The most numerous and intense zones of antioxidant activity in this group of extracts was shown by *Ga* L MeOH:H_2_O extract. More zones of antioxidant activity were shown when *Galium verum* plant materials were analyzed. In this case, the freeze-dried plant material extracted in a mixture of methanol:water (70:30 *v*/*v*) was also characterized by the occurrence of the most numerous and intense zones of antioxidant activity. Analysis of extracts from *Solidago virgaurea* and *Solidago canadensis* showed the strongest antioxidant activity, where the use of methanol:water extracts from freeze-dried plant materials once again provided the best results. All extracts showed the presence of chlorogenic acid (Rf about 0.42). In addition, all extracts except *Galium aparine* also showed the presence of rutin (Rf approx. 0.26). The complete results of HPTLC-DPPH^•^ analysis are shown in Figure 1 and Figure 2.

### 2.4. HPTLC-Chymotrypsin Inhibition

**Figure 3:**
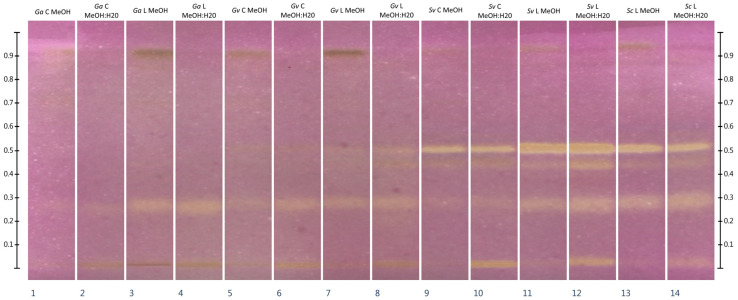
Results of HPTLC-Chymotrypsin inhibition analysis of methanolic and methanol:water extracts. Samples were applied at volume of 10 µL. MeOH—methanolic extracts, MeOH:H_2_O—extracts prepared in methanol:water mixture (70:30 *v*/*v*), *Ga*—*Galium aparine* L., *Gv*—*Galium verum* L., *Sv*—*Solidago virgaurea* L., *Sc*—*Solidago canadensis* L., C—extracts from commercially available plant material, L—extracts from freeze-dried plant material self-collected from wild sites. Stationary phase: HPTLC silica gel 60 plates on glass support (20 × 10 cm). Development conditions: first development in MP 4.1: ethyl acetate:methyl ethyl ketone:methanol:diethyl ether:water (30:30:10:10:10 *v*/*v*/*v*/*v*/*v*) at distance of 5 cm from application zone, second development in MP 4.2: chloroform:ethyl acetate (40:10 *v*/*v*) to the height of the target solvent front position (70 mm from the application line). Substrate (BApNA) at a concentration of 11 mM was applied first; then, the enzyme solution in buffer (3 mg/mL) was applied to the plate by spraying. Derivatization agents: 0.05% (*w*/*v*) solution of NPED in water (reagent 1) followed by 0.5% (*w*/*v*) solution of NaNO_2_ in 1.2 M HCl (reagent 2). The plate was incubated in a sealed plastic container lined with a moistened paper towel, at room temperature, in the dark, for one hour.

**Figure 4:**
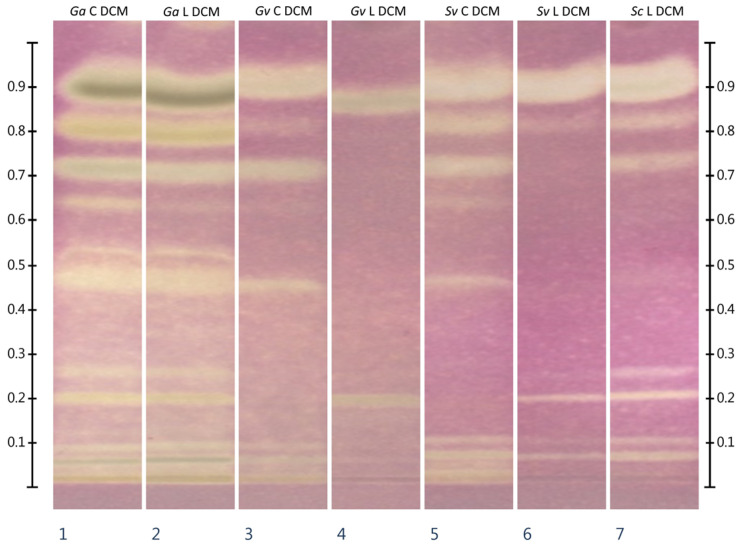
Results of HPTLC-Chymotrypsin inhibition analysis of dichloromethane extracts. Samples were applied at volume of 20 µL. DCM—extracts prepared in dichloromethane, *Ga*—*Galium aparine* L., *Gv*—*Galium verum* L., *Sv*—*Solidago virgaurea* L., *Sc*—*Solidago canadensis* L., C—extracts from commercially available plant material, L—extracts from freeze-dried plant material self-collected from wild sites. Stationary phase: HPTLC silica gel 60 plates on glass support (20 × 10 cm). Development conditions: MP 3: ethyl acetate:toluene (80:20 *v*/*v*) to the height of 70 mm from the application line. Substrate (BApNA) at a concentration of 11 mM was applied first; then, the enzyme solution in buffer (3 mg/mL) was applied to the plate by spraying. Derivatization agents: 0.05% (*w*/*v*) solution of NPED in water (reagent 1) followed by 0.5% (*w*/*v*) solution of NaNO_2_ in 1.2 M HCl (reagent 2). The plate was incubated in a sealed plastic container lined with a moistened paper towel, at room temperature, in the dark, for one hour.

Effect-directed analysis of the inhibitory activity of extracts (methanolic and methanol:water) from commercial *Galium aparine* plant material against chymotrypsin showed the presence of a single, barely detectable zone of inhibition (Rf about 0.27). For extracts from freeze-dried *G. aparine*, the presence of a significantly more intense zone of inhibition was observed, with the same Rf value. Extracts from commercial *Galium verum* plant material showed inhibitory activity intermediate between commercial and freeze-dried *Galium aparine* at this Rf value. In the case of the freeze-dried *Galium verum*, in addition to the presence of an inhibition zone at an Rf of about 0.27, there was also a slight inhibitory activity zone at an Rf about 0.50. In the case of the methanol extract from the commercial *Solidago virgaurea*, one moderately strong inhibition zone at an Rf of approximately 0.50 and two weak ones at Rf of about 0.27 and 0.45 are evident. In the case of methanol:water extract from the same raw material, in conjunction with the aforementioned zones of inhibition, there was also a zone at an Rf of about 0.00 (application line). Zones of enzyme inhibition for methanol and methanol:water extracts from freeze-dried *Solidago virgaurea* and *S. canadensis* occurred at analogous Rf values to those of *Sc* C extracts, but were characterized by greater intensity. Different results were obtained when analyzing the inhibitory activity of dichloromethane extracts. In this case, the most numerous and intense zones of inhibition were observed in the presence of extracts from commercial and freeze-dried *Galium aparine* followed by commercial *S. virgaurea*, freeze-dried *S. canadensis* and commercial *G. verum* plant materials. The least numerous zones of chymotrypsin inhibition were observed for freeze-dried *Solidago virgaurea* and *Galium verum* dichloromethane extracts. The complete HPTLC-Chymotrypsin inhibition results are shown in Figure 3 and Figure 4.

## 3. Discussion

The total concentrations of phenolic compounds and flavonoids in all analyzed samples were greater for plant materials obtained from proprietary collections of wild sites that were subjected to lyophilization, as compared to commercially available materials, which were likely air-dried. This observation aligns with findings by Senio et al. [30], whose results denote a higher concentration of phenolic compounds in extracts derived from lyophilized plant materials. In this study, freeze-dried plants were compared with commercially available samples. It is therefore possible that, in addition to the freeze-drying process itself, other factors also influenced the differences in antioxidant and chymotrypsin inhibitory activity. These include, among others, potential harvesting at a different stage of growth, and plant development in different atmospheric and habitat conditions. Methanol extracts exhibited a greater total concentration of flavonoids relative to methanol:water extracts (70:30 *v*/*v*). Conversely, with regard to the total phenolics content, the trend was reversed, as methanol:water extracts demonstrated higher TPC values. The increased TPC in lyophilizates predominantly correlated with elevated antioxidant activity values when juxtaposed with extracts from commercially available plant materials. An exception was noted with extracts derived from *Galium verum* in the DPPH^•^ and ABTS^+•^ assays, where the values for extracts from both freeze-dried and commercial materials were comparable. Furthermore, the antioxidant activity of all dichloromethane extracts was significantly inferior to that of methanol and methanol:water extracts.

To the best of the authors’ knowledge, the analyzed plant materials have not previously been subjected to HPTLC-DPPH^•^ analysis. The analyses performed, although they do not provide quantitative information about total antioxidant activity of extracts, correspond to some extent with the results of total antioxidant activity in UV-Vis tests. Analysis of dichloromethane extracts from freeze-dried raw materials showed the presence of one intensive zone of antioxidant activity for each analyzed extract. Intensive inhibition zones were not observed in the case of extracts from commercial dried materials. Analysis of methanolic and methanol:water extracts also correspond with the results of total antioxidant activity in tests using UV-Vis spectrophotometry. Visual evaluation of chromatograms after derivatization with the DPPH^•^ reagent indicates the occurrence of the most numerous and most intensive inhibition zones in raw materials obtained from *Solidago* sp. The fewest activity zones could be observed in the case of extracts obtained from *Galium aparine* plant materials, while *Galium verum* extracts show intermediate activity. A greater number of more intense inhibition zones can also be observed in the case of extracts from freeze-dried raw materials, compared to commercial ones. Also, the tracks corresponding to methanol:water extracts contain a greater number of more intense zones of antioxidant activity than those corresponding to methanol extracts. The use of rutin and chlorogenic acid standards in the analysis also showed the presence of chlorogenic acid in all the extracts analyzed. Rutin, on the other hand, was present in all extracts except those obtained from *Galium aparine*.

To the best of the authors’ knowledge, there are no reports of inhibitory activity of *Galium aparine*, *Galium verum*, *Solidago virgaurea* and *Solidago canadensis* against the enzyme α-chymotrypsin. The results of effect-directed HPTLC analysis showed the presence of numerous chymotrypsin inhibitors in dichloromethane extracts of *Galium aparine*, *Solidago virgaurea*, and *Solidago canadensis*. The most numerous and intense inhibition zones were observed in extracts obtained from *Galium aparine*, regardless of whether lyophilized or commercially available material was used. In the case of *Galium verum* and *Solidago virgaurea*, the commercial material showed a greater number of chymotrypsin inhibition zones than the lyophilized material. The dichloromethane extract from the freeze-dried *Solidago canadensis* plant material showed a similar number and intensity of inhibition zones as the commercial *Solidago virgaurea* plant material extract, although some zones were other chemical compounds (occurring at different Rf values). Analysis of methanol and methanol:water extracts showed negligible inhibitory activity of the extracts from the commercial *Galium aparine* plant material. In the case of the freeze-dried material from this plant, the obtained inhibition zones at Rf of approx. 0.27 were more intense. In the case of *Galium verum* extracts, a similar property was observed—application of extracts obtained from the freeze-dried material was reflected in a higher intensity of inhibition zones. The same property was also observed in the case of *Solidago virgaurea* extracts. In all methanol:water extracts of *Solidago* sp., a zone of enzymatic inhibition could be observed at the height of the application line (Rf~0), which was not present in the methanolic extracts. Despite the many advantages of using effect-directed high-performance thin-layer chromatography (ED-HPTLC), there are also some limitations. In particular, this technique does not provide information on the structure of compounds exhibiting the observed direction of biological activity. Further analyses are necessary to determine the structure of the inhibitor.

Freeze-drying seems to be an effective approach for preserving plant material with regard to maximizing the retention of phenolic compounds. The analyses performed on dichloromethane extracts showed higher antioxidant activity of extracts from freeze-dried plant material, both in spectrophotometric tests and HPTLC-DPPH^•^. In the chymotrypsin inhibition test, the trend turned out to be the opposite, and extracts from commercially available plant material used in the procedure resulted in the presence of a greater number of enzymatic inhibition zones. The obtained results indicate the potential of using extracts from wild herbaceous plants, commonly treated as weeds, as nutraceuticals and pharmaceuticals. Moreover, it was shown that the antioxidant and chymotrypsin inhibitory activity of the invasive species *Solidago canadensis* is similar to *Solidago virgaurea*. The obtained results confirm the need for further exploration of native plants, often treated as weeds, using modern analytical techniques, because they can constitute an interesting matrix for the isolation of biologically active substances.

## 4. Materials and Methods

### 4.1. Chamicals and Reagents

α-Chymotrypsin from bovine pancreas (type-II, lyophilized powder, ≥40 units/mg protein), Nα-Benzoyl-DL-arginine 4-nitroanilide hydrochloride (BApNA), sodium nitrite (crystals for synthesis), Triton™ X-100 (for molecular biology), tris(hydroxymethyl)amino-methane (tris base), N-(1-Naphthyl)ethylenediamine dihydrochloride (NPED), 2,2-Diphenylpicrylhydrazyl (DPPH), gallic acid, rutin, chlorogenic acid, iron (III) chloride, 2,2′-Azino-bis(3-ethylbenzothiazoline-6-sulfonic acid) (ABTS), potassium persulfate, aluminum (III) chloride, Folin & Ciocâlteu phenol reagent (F-C), 6-Hydroxy-2,5,7,8-tetramethylchroman-2-carboxylic Acid (Trolox), 2,4,6-Tris(2-pyridyl)-s-triazine (TPTZ), sodium acetate, sodium carbonate, HPTLC silica gel 60 plates on aluminum and glass support were purchased from Sigma-Aldrich (Saint Louis, MO, USA). Methanol (LC-MS grade) was purchased from Merck (Darmstadt, Germany). Ethyl acetate (p.a.) was purchased from Honeywell (Morristown, NJ, USA). Dimethyl sulfoxide (DMSO, p.a.), chloroform (p.a) and dichloromethane (p.a.) were purchased from Stanlab (Lublin, Poland). Diethyl ether (p.a.) and formic acid (p.a.) were purchased from POCH (Gliwice, Poland) and 2-butanone (p.a.) was purchased from Chempur (Piekary Śląskie, Poland). Ultrapure (type 1) water was produced in Synergy^®^ UV device (Merck, Darmstadt, Germany). VisionCATS software v.2.5 (Muttenz, Switzerland) was used in the HPTLC procedure.

### 4.2. Procedure Diagram

**Figure 5:**
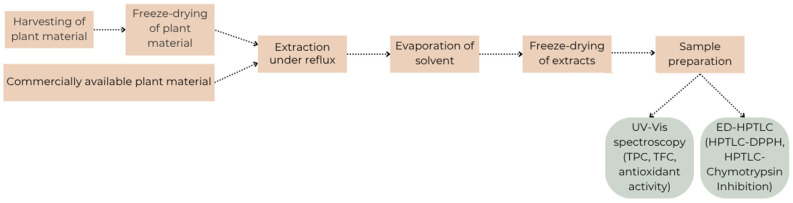
Schematic procedure.

The procedure followed during the study is presented in Figure 5.

### 4.3. Plant Material

The aerial parts of *Galium aparine* L. (during flowering, before fruit formation) were collected in Opole, Opole Voivodeship, Poland (May 2024). The aerial parts of *Galium verum* L. and *Solidago virgaurea* L. (during flowering stage) were collected in Grzybowo, West Pomeranian Voivodeship, Poland (August 2024). The aerial parts of *Solidago canadensis* L. (during flowering stage) were collected in Opole, Opole Voivodeship, Poland (August 2024). The whole plants was freeze-dried at −50 °C for 48 h instantly after harvest (freeze-dryer ChristAlpha 1-2 LDplus, Osterode am Harz, Germany). The preserved plant material was stored at room temperature in airtight containers in a shaded place until further analysis. Commercially available plant materials of *Galium aparine* L., *Galium verum* L. and *Solidago virgaurea* L. were also included in the analysis.

### 4.4. Extraction and Preparation of Crude Extracts

Freeze-dried aerial parts of plants and commercially available samples were subjected to extraction under reflux. For this purpose, 5 g of powdered plant material was extracted with 100 cm^3^ of methanol, methanol:water mixture (70:30 *v*/*v*) or dichloromethane. After an hour of extraction in boiling solvent, the extract was separated from the precipitate by vacuum-assisted filtration. The remaining plant material was subjected to re-extraction for an hour in 50 cm^3^ of the same solvent. The extract was filtered again. Extracts of the same type were combined. Each of the extracts was then dried in a vacuum evaporator (Rotovapor R-100, Buchi, Flawil, Switzerland) at 35 ± 1 °C. Then, the dried extracts were freeze-dried (under the same conditions as for freeze-drying of plant material) to remove residues. Dry extracts were weighed to determine extraction efficiency and then stored in airtight vials in the freezer (about −20 ± 2 °C) until further analysis.

### 4.5. UV-Vis Spectrophotometric Assays

Total phenolics content (TPC, in assay with Folin & Ciocâlteu phenol reagent), total flavonoids content (TFC, in assay with AlCl_3_) and total antioxidant activity (in tests with DPPH^•^, ABTS^+•^ and FRAP reagents) were measured using spectrophotometric assays (Hitachi U-2810 spectrophotometer, Tokyo, Japan).

#### 4.5.1. Total Phenolic Content (TPC)

TPC determinations were performed based on the methodology described in the publication by Öztürk Ş. et al. [31] with minor modifications. TPC results were expressed as gallic acid equivalent in mg/g of crude extract. Disposable, plastic spectrophotometric cuvettes with a light path length of 1 cm were used for the measurements. The standard curve was calculated based on the concentration of gallic acid ranging from 0.005 to 0.025 mg/mL. The maximum absorbance of the complex was measured at a wavelength of 750 ± 5 nm. Crude methanolic extracts were dissolved in methanol, while methanol:water extracts were dissolved in methanol:water (70/30 *v*/*v*). The solutions were sonicated for 10 min (Elmasonic S 30 H, Elma, Singen, Germany). The concentrations of all extract solutions used in the TPC analysis were 5 mg/mL, except for *Sc* L MeOH and MeOH:H_2_O (2.5 mg/mL). Initially, 0.1 mL of F-C reagent was added to 1.5 mL of HPLC grade water, followed by 0.1 mL of the extract solution of the above concentration. After 6 min, 0.3 mL of 20% Na_2_CO_3_ solution was added to the mixture. The samples were left for 2 h in the dark space and then subjected to absorbance measurement. In the case of the blank sample, the F-C reagent was replaced by an equivalent volume of HPLC grade water (0.1 mL). All measurements were repeated three times (N = 3), and the results were presented as an arithmetic mean ± Standard Deviation (SD) of the obtained measurements.

#### 4.5.2. Total Flavonoids Content (TFC)

TFC determinations were performed according to the methodology described by Chandra S. et al. [32] with minor modifications. TFC results were expressed as rutin equivalent in mg/g of crude extract. Disposable, plastic spectrophotometric cuvettes with a light path length of 1 cm were used for the measurements. The standard curve was calculated based on the concentration of rutin ranging from 0.002778 to 0.033333 mg/mL. The absorbance maximum for the complex was measured at a wavelength of 425 ± 5 nm. Crude methanolic extracts were dissolved in methanol, while methanol:water extracts were dissolved in methanol:water (70/30 *v*/*v*), and the solutions were sonicated for 10 min. The concentrations of the extracts solutions used in the TFC analysis were 5 mg/mL for *Ga* C MeOH, *Ga* L MeOH, *Gv* C MeOH, *Gv* L MeOH, *Sv* C MeOH; 10 mg/mL for *Ga* L MeOH:H_2_O and *Gv* C MeOH:H_2_O; 7.5 mg/mL for *Gv* L MeOH:H_2_O, *Sv* L MeOH, *Sv* L MeOH:H_2_O and *Sc* L MeOH:H_2_O; and 2.5 mg/mL for *Sc* L MeOH. To 1.5 mL of methanol, 0.5 mL of the extract solution of the above concentration was added, followed by 0.25 mL of 2% AlCl_3_ solution. After an hour of incubation at room temperature, the maximum absorbance of the obtained complexes was measured. In the case of the blank sample, the AlCl_3_ solution was replaced by an equivalent volume of HPLC grade water (0.25 mL). All measurements were repeated three times (N = 3), and the results were presented as the arithmetic mean ± Standard Deviation (SD) of the obtained measurements.

#### 4.5.3. Antioxidant Activity—DPPH^•^

Total antioxidant activity tests with the DPPH^•^ radical were performed based on the methodology described by Sielicka M. et al. [33] with minor changes. A fresh methanolic DPPH^•^ solution (2.5 mg/100 mL) was prepared, and its absorbance value was corrected (by adding DPPH^•^ or methanol) to 0.700 ± 0.050 at a wavelength of 515 nm. The results of antioxidant activity in the DPPH^•^ test were expressed as Trolox equivalent in mg/g of crude extract. Disposable, plastic spectrophotometric cuvettes with a light path length of 1 cm were used for measurements of methanol and methanol:water (70:30 *v*/*v*) extracts, and quartz cuvettes, also with a light path length of 1 cm, were used for measurements of dichloromethane extracts. The standard curve was calculated based on the concentration of Trolox ranging from 0.000 (without Trolox) to 0.005 mg/mL. Crude methanolic extracts were dissolved in methanol, methanol:water extracts in methanol:water (70/30 *v*/*v*) and dichloromethane extracts in dichloromethane. The solutions were sonicated for 10 min. The concentrations of the extract solutions used in the DPPH^•^ test were 1 mg/mL for *Ga* C MeOH and MeOH:H_2_O, *Ga* L MeOH and MeOH:H_2_O, *Gv* C MeOH and MeOH:H_2_O, *Gv* L MeOH and MeOH:H_2_O, *Sv* C MeOH and MeOH:H_2_O and *Sc* L MeOH. Extract concentrations of 0.5 mg/mL were used for *Sv* L MeOH and MeOH:H_2_O and *Sc* L MeOH:H_2_O. For dichloromethane extracts, the concentration of all extract solutions were 2 mg/mL. To 1.95 mL of DPPH^•^ solution, 0.05 mL of the extract solutions with the above concentrations were added. After 30 min of incubation in the dark space at room temperature, the absorbance of the extracts was measured at a wavelength of 515 ± 1 nm. All measurements were repeated three times (N = 3), and the results were presented as an arithmetic mean ± Standard Deviation (SD) of the obtained measurements.

#### 4.5.4. Antioxidant Activity—FRAP

The FRAP assay was performed based on the methodology described by Szôllôsi R. et al. [34] with minor modifications. The FRAP reagent was obtained by mixing 100 mL of 0.3 M acetate buffer pH 3.6 (at 37 °C) with 10 mL of 10 mM TPTZ in 40 mM HCl and 20 mM FeCl_3_ aqueous solution. The absorbance value of the FRAP reagent was corrected to 0.200 ± 0.020 at a wavelength of λ = 593 nm by adding HPLC grade water. The results of antioxidant activity in the FRAP assay were expressed as Trolox equivalent in mg/g of crude extract. Disposable, plastic spectrophotometric cuvettes with a light path length of 1 cm were used for the measurements. The standard curve was calculated based on the concentration of Trolox ranging from 0.00000 (without Trolox) to 0.00625 mg/mL. Crude methanolic extracts were dissolved in methanol, while methanol:water extracts were dissolved in methanol:water (70:30 *v*/*v*). The solutions were sonicated for 10 min. The concentrations of the extract solutions used in the FRAP assay were 1 mg/mL except for *Sv* L MeOH and MeOH:H_2_O and *Sc* L MeOH:H_2_O (0.5 mg/mL). To 1.95 mL of the FRAP reagent, 0.05 mL of the extract solutions with the above concentrations was added. After 4 min of incubation in the dark space at 37 °C, the absorbance of the extracts was measured at a wavelength of 593 ± 2 nm. All measurements were repeated three times (N = 3), and the results were presented as an arithmetic mean ± Standard Deviation (SD) of the obtained measurements.

#### 4.5.5. Antioxidant Activity—ABTS^+•^

ABTS^+•^ assay was performed based on the methodology described by Lee K.J. et al. [35] with minor modifications. ABTS^+•^ cation radical was generated by mixing 5 mL of 7 mM ABTS solution with 88 µL of 140 mM potassium persulfate solution (ABTS^+•^ stock solution). The mixture was left at room temperature in the dark space for 16 h. Then 1 mL of ABTS^+•^ stock solution was mixed with 100 mL of methanol. The absorbance value of ABTS^+•^ reagent was corrected to 0.700 ± 0.050 at λ = 745 nm by adding methanol or ABTS^+•^ stock solution. Disposable plastic spectrophotometric cuvettes with a light path length of 1 cm were used for measurements of methanolic and methanol:water (70:30 *v*/*v*) extracts and quartz cuvettes with a light path length of 1 cm were used for measurements of dichloromethane extracts. The standard curve was calculated based on the concentration of Trolox ranging from 0.000 (without Trolox) to 0.005 mg/mL. Crude methanolic extracts were dissolved in methanol, methanol:water extracts in a methanol:water mixture (70:30 *v*/*v*), and dichloromethane crude extracts in dichloromethane. The extract solutions were sonicated for 10 min. The concentrations of the extract solutions used in the ABTS^+•^ test were 1 mg/mL for *Ga* C MeOH and MeOH:H_2_O, *Gv* L MeOH and MeOH:H_2_O and *Sc* L MeOH. The concentrations of the extracts were 0.5 mg/mL for *Gv* C MeOH and MeOH:H_2_O, *Ga* L MeOH and MeOH:H_2_O, *Sc* L MeOH:H_2_O, and *Sv* L MeOH and MeOH:H_2_O. The extracts at a concentration of 0.25 mg/mL were used in the determination of the antioxidant activity of the extracts: *Sv* C MeOH and MeOH:H_2_O. In the case of dichloromethane extracts, the concentrations of all solutions were 2 mg/mL. To 1.95 mL of ABTS^+•^ reagent, 0.05 mL of the extract solutions at the concentrations given above were added. After 6 min of incubation in the dark space at room temperature, the absorbance of the extracts was measured at a wavelength of 745 ± 1 nm. All measurements were repeated three times (N = 3), and the results were presented as an arithmetic mean ± Standard Deviation (SD) of the obtained measurements.

### 4.6. HPTLC

HPTLC analysis was conducted using CAMAG Linomat 5, CAMAG Visualizer 3 and VisionCATS software v.2.5 (Muttenz, Switzerland). The general conditions of the analysis were as follows: plate format—20 × 10 cm, application type—band, position Y—8.0 mm, length—8.0 mm, width—0.0 mm, first track position X—14.5 mm, distance—11.4 mm. The dosing speed was set to 80 nL/s for methanolic and methanol:water extracts (70:30 *v*/*v*) and 200 nL/s for dichloromethane extracts. Pre-dosage volume was set to 0.20 µL. The stationary phase was HPTLC plates silica gel 60 on an aluminum backing (except for the analyses of chymotrypsin inhibitory activity for which plates on a glass backing were used). The plates were developed to a height of 70 mm from the sample application line (78 mm from the bottom edge of the plate) in a 20 × 20 cm glass chamber filled with 100 mL of mobile phase after a saturation time of 20 min. The concentrations of the applied extracts were 25 mg/mL for methanol and methanol:water extracts (70:30 *v*/*v*) and 5 mg/mL for dichloromethane extracts. Crude methanolic extracts were dissolved in methanol, methanol:water in a methanol:water mixture (70:30 *v*/*v*), and dichloromethane extracts in dichloromethane. The solutions were sonicated for 10 min. Then the solutions were filtered through a syringe filter (PURELAND, 0.45 µm, Nylon). Before analysis, plates were pre-developed along the entire length of the plate (10 cm) in methanol and dried at 110 °C for 10 min (Memmert UF110, Schwabach, Germany) to remove contaminants and bound moisture.

#### 4.6.1. HPTLC-DPPH^•^

The HPTLC-DPPH^•^ assay methodology was loosely based on the publication by Islam K. et al. [36]. Extracts were applied to Silica gel 60 HPTLC plates on an aluminum support (20 × 10 cm) pre-developed as described above in the following quantities: all dichloromethane extracts were applied in a quantity of 75 µg, *Solidago* sp. methanolic and methanol:water extracts were applied in a quantity of 62.5 µg and *Galium* sp. MeOH and MeOH:H_2_O extracts were applied in a quantity of 75 µg. Plates with dichloromethane extracts were developed in the following mobile phase: ethyl acetate:toluene:formic acid (80:20:2 *v*/*v*/*v*, MP 1). Plates with methanol and methanol:water extracts were developed in two successive mobile phases: ethyl acetate:methyl ethyl ketone:methanol:diethyl ether:water:formic acid (30:30:10:10:10:10 *v*/*v*/*v*/*v*/*v*/*v*, MP 2.1), which then developed into the second system of chloroform:ethyl acetate:formic acid (40:10:1 *v*/*v*/*v*, MP 2.2). After each development, the plates were dried at 110 °C for 10 min. In the case of development in MP 2, the degree of separation can be controlled by selecting the height to which the plate was developed in MP 2.1. In MP 2.2, the plate was developed to the height of the target solvent front position (70 mm from the application line). After each development and drying step, the plates were photographed in white light, UV 254 nm and 366 nm. The developed chromatograms were derivatized with DPPH^•^ methanolic solution (2.5 mM) by uniformly spraying the plate with 5 mL of the reagent. After DPPH^•^ application, the plates were incubated for 30 min in a dark place, and then, the plates were photographed in white light (white remission and white remission + white transmission). The zones of antioxidant activity were revealed as yellow bands on a pink-purple background.

#### 4.6.2. HPTLC-Chymotrypsin Inhibition

The procedure for determining chymotrypsin inhibitory activity in effect-directed HPTLC was based on the publication by Legerská B. et al. [37] with modifications. The enzyme was dissolved in 3 mg/mL of 0.1 M Tris-HCl buffer pH 8.0, as specified in the protocol. The substrate for the enzyme (BApNA) was dissolved in 12.5% DMSO in methanol with the addition of 0.1% Triton™ X-100, which allowed for increasing the solubility of the substrate. BApNA was used at a concentration of 11 mM. The modified Griess method was used as the derivatization technique, according to the protocol. For this purpose, immediately before derivatization, a 0.05% (*w*/*v*) solution of NPED in water (reagent 1) and a 0.5% (*w*/*v*) solution of NaNO_2_ in 1.2 M HCl (reagent 2) were prepared. Dissolution of the compounds was assisted by sonication in an ultrasonic bath for 5 min. The acid additive was eliminated from the mobile phases used in the procedure. In the case of dichloromethane extracts, the ethyl acetate:toluene (80:20 *v*/*v* MP 3) was used. Plates with methanol and methanol:water extracts were developed in two successive mobile phases: ethyl acetate:methyl ethyl ketone:methanol:diethyl ether:water (30:30:10:10:10 *v*/*v*/*v*/*v*/*v*, MP 4.1), which then developed into the second system of chloroform:ethyl acetate (40:10 *v*/*v*, MP 4.2). The order of applying the derivatization agents was changed in relation to the original protocol—the NPED solution was applied first, and then, after drying in a stream of warm air, NaNO_2_ in 1.2 M HCl was applied. The applied modifications enhanced the visual contrast of the light enzymatic inhibition zones against the pink background.

The extracts were applied to silica gel 60 HPTLC plates on a glass backing (20 × 10 cm) pre-developed (as described in the HPTLC general procedure) in the following quantities: all dichloromethane extracts were applied at a quantity of 100 µg and all methanolic and methanol:water extracts were applied at a quantity of 250 µg. Plates with dichloromethane extracts were developed in MP 3 to a height of 70 mm from the sample application line. Chromatograms with MeOH and MeOH:H_2_O extracts were developed in MP 4.1 and then MP 4.2. In the case of development in MP 4, the degree of separation can be controlled by selecting the solvent front position to which the plate was developed in MP 4.1. In MP 4.2, the plate was developed to the target solvent front position (70 mm from the application line). After each development stage, the plates were dried at 110 °C for 10 min. After each development and drying stage, photographs of the plates were taken in white light, UV 254 nm, and 366 nm. Then, 7 mL of BApNA solution was uniformly applied to the plates by spraying, and the plate was dried in a stream of warm air. The enzyme solution in buffer (7 mL) was evenly applied to the plate with the substrate by spraying. The plate was incubated in a sealed plastic container lined with a moistened paper towel, at room temperature, in the darkness, for one hour. After the incubation period, the plate was dried in a stream of warm air until dry. Subsequently, 7 mL of derivatization reagent 1 was applied to the plate by atomization, allowed to dry, and then 7 mL of derivatization reagent 2 was applied in the same manner and photos of the resulting bright fields of enzyme inhibition on a dark pink background were taken in white light remission, remission + transmission and transmission.

## Figures and Tables

**Table 1:** Crude extract weight [mg of crude extract per gram of plant material] after extraction of 5 g of plant material and evaporation of solvent followed by freeze-drying.

Extract Type	Extract Yield from Plant Material [mg/g]		
	Methanol	Methanol:Water (70:30 *v*/*v*)	Dichloromethane
*G. aparine* commercial	116.64	162.12	10.86
*G. aparine* lyophilized	147.08	179.22	10.74
*G. verum* commercial	130.38	160.46	17.10
*G. verum* lyophilized	188.08	194.08	15.78
*S. virgaurea* commercial	117.78	218.88	17.12
*S. virgaurea* lyophilized	128.82	266.70	15.34
*S. canadensis* lyophilized	214.68	315.40	15.66

**Table 2:** Results of total phenolics, total flavonoids, and total antioxidant activity spectrophotometric assays.

Total Phenolics Content (TPC), Total Flavonoids Content (TFC) and Antioxidant Activity												
Plant Extract	TPC [mg GAE/g Crude Extract]		TFC [mg RE/g Crude Extract]		DPPH^•^ [TE/g Crude Extract]			FRAP [TE/g Crude Extract]		ABTS^+•^ [TE/g Crude Extract]		
	MeOH	MeOH:H_2_O	MeOH	MeOH:H_2_O	MeOH	MeOH:H_2_O	DCM	MeOH	MeOH:H_2_O	MeOH	MeOH:H_2_O	DCM
*Ga* C	34.49 ± 1.12	37.18 ± 0.00	1.61 ± 0.09	0.16 ± 0.04	33.73 ± 3.57	50.76 ± 2.96	19.55 ± 1.51	17.48 ± 0.28	18.13 ± 0.51	32.71 ± 3.43	41.46 ± 1.62	18.68 ± 1.22
*Ga* L	56.73 ± 2.36	60.10 ± 2.70	0.74 ± 0.13	0.35 ± 0.04	78.36 ± 3.91	110.87 ± 4.44	27.07 ± 0.93	57.70 ± 0.61	67.31 ± 2.08	69.31 ± 4.05	109.69 ± 2.99	27.96 ± 0.37
*Gv* C	56.00 ± 1.12	84.62 ± 2.54	4.45 ± 0.33	1.67 ± 0.04	71.15 ± 4.96	122.37 ± 1.84	15.33 ± 0.35	49.15 ± 2.63	88.31 ± 2.19	75.79 ± 2.33	112.28 ± 7.56	11.39 ± 0.93
*Gv* L	76.04 ± 1.27	122.75 ± 0.00	6.32 ± 0.16	2.68 ± 0.15	66.55 ± 1.06	126.05 ± 1.66	20.17 ± 2.26	75.12 ± 0.56	144.41 ± 0.28	49.01 ± 2.94	118.76 ± 3.11	21.70 ± 0.28
*Sv* C	93.15 ± 5.50	129.11 ± 1.69	2.53 ± 0.49	1.47 ± 0.35	124.97 ± 3.91	177.42 ± 5.79	20.17 ± 1.70	107.52 ± 1.74	134.72 ± 4.44	149.85 ± 4.55	171.44 ± 8.23	12.47 ± 0.58
*Sv* L	184.89 ± 6.11	200.53 ± 4.48	4.34 ± 0.00	2.33 ± 0.12	163.47 ± 3.72	285.22 ± 4.22	17.17 ± 2.06	183.13 ± 8.00	294.84 ± 6.11	142.29 ± 1.98	249.39 ± 9.34	21.43 ± 1.10
*Sc* L	155.02 ± 1.47	157.95 ± 6.39	4.72 ± 0.19	3.93 ± 0.11	152.43 ± 1.62	218.98 ± 5.75	24.77 ± 3.14	169.40 ± 3.67	234.75 ± 3.10	125.56 ± 5.53	194.98 ± 8.91	25.96 ± 1.13

MeOH—methanolic extracts; MeOH:H_2_O—extracts prepared in methanol:water mixture (70:30 *v*/*v*); DCM—extracts prepared in dichloromethane; GAE—gallic acid equivalent; RE—rutin equivalent; TE—Trolox equivalent, *Ga*—*Galium aparine* L., *Gv*—*Galium verum* L., *Sv*—*Solidago virgaurea* L., *Sc*—*Solidago canadensis* L., C—extracts from commercially available plant material, L—extracts from freeze-dried plant material self-collected from wild sites. The data were presented in the following format: arithmetic mean of 3 measurements (N = 3) ± Standard Deviation (SD).

## Data Availability

The data presented in this study are available on request from the corresponding author.

## Abbreviations

The following abbreviations are used in this manuscript:

Ga C	Galium aparine L. commercial plant material
Ga L	Galium aparine L. lyophilized plant material self-collected from wild sites
Gv C	Galium verum L. commercial plant material
Gv L	Galium verum L. lyophilized plant material self-collected from wild sites
Sv C	Solidago virgaurea L. commercial plant material
Sv L	Solidago virgaurea L. lyophilized plant material self-collected from wild sites
Sc L	Solidago canadensis L. lyophilized plant material self-collected from wild sites
